# Continuous Flow Photocatalytic Hydrogen Production from Water Synergistically Activated by TiO_2_, Gold Nanoparticles, and Carbon Nanotubes

**DOI:** 10.3390/nano13071184

**Published:** 2023-03-27

**Authors:** Joseph Farah, Florent Malloggi, Frédéric Miserque, Jongwook Kim, Edmond Gravel, Eric Doris

**Affiliations:** 1 Université Paris-Saclay, CEA, CNRS, NIMBE, 91191 Gif-sur-Yvette, France; 2 Université Paris-Saclay, Département Médicaments et Technologies pour la Santé (DMTS), CEA, INRAE, SCBM, 91191 Gif-sur-Yvette, France; 3 Université Paris-Saclay, Service de Recherche en Corrosion et Comportement des Matériaux, CEA, 91191 Gif-sur-Yvette, France; 4 Laboratoire de Physique de la Matière Condensée, Ecole Polytechnique, CNRS, IP Paris, 91128 Palaiseau, France

**Keywords:** hydrogen production, titanium dioxide, carbon nanotube, gold nanoparticles, flow chemistry

## Abstract

Titanium dioxide nanoparticles were combined with carbon nanotubes and gold to develop improved photocatalysts for the production of hydrogen from water. The entangled nature of the nanotubes allowed for the integration of the photoactive hybrid catalyst, as a packed-bed, in a microfluidic photoreactor, and the chips were studied in the photocatalyzed continuous flow production of hydrogen. The combination of titanium dioxide with carbon nanotubes and gold significantly improved hydrogen production due to a synergistic effect between the multi-component system and the stabilization of the active catalytic species. The titanium dioxide/carbon nanotubes/gold system permitted a 2.5-fold increase in hydrogen production, compared to that of titanium dioxide/carbon nanotubes, and a 20-fold increase, compared to that of titanium dioxide.

## 1. Introduction

In the context of renewable energies, hydrogen gas is regarded not only as a clean and sustainable carrier, but also as the main alternative to carbon-based fuels [1]. While most molecular hydrogen is currently produced from natural gas through the reforming of methane, it can also be obtained in a more sustainable manner from renewable sources [2], such as water. In fact, water constitutes an ideal raw material [3] to cleanly generate H_2_, with minimal impact on the environment. Water can be used to produce hydrogen gas by electro-catalytic reduction (hydrogen evolution reaction—HER), which involves platinum electrodes in most industrial electrolytic cell systems. On the other hand, photocatalytic water splitting [4] represents an interesting alternative, as conversion of light into chemical energy is practical, and sunlight is the largest renewable energy source. 

Pioneering work in the field of the photocatalytic production of molecular hydrogen from water dates from 1972, with the seminal publication of Fujishima and Honda who reported the first use of titanium dioxide (TiO_2_) in a photo-electrochemical cell [5]. TiO_2_ has since been widely explored to catalytically convert H_2_O to H_2_ by photo-activation. The photo-activated process generates electron–hole pairs that are capable of performing reduction and oxidation reactions at the surface of the semi-conductor nanomaterial [6].

Although effective, TiO_2_ suffers from drawbacks related to its band gap energy, which lies in the UV region (ca. 3.2 eV), and to charge recombination, which decreases the overall photocatalytic performance. The charge recombination phenomenon can be minimized by associating the active semi-conductor material (i.e., TiO_2_) with a metallic nanoparticle co-catalyst, which allows for charge migration and separation [7], while providing additional reaction sites. In addition, charge dissociation has also been evidenced upon association of TiO_2_ with carbonaceous platforms, such as carbon nanotubes (CNT) [8]. This interaction also slows down the recombination of photo-induced carriers through the formation of heterojunctions [9]. On the other hand, visible light activation of TiO_2_-based material can be achieved by sensitization with a dye [10], or by combination with noble metal (e.g., gold) nanoparticles (NP) [11], whose localized surface plasmon resonance can be activated outside the UV region. 

With these features in mind, and as part of our long-standing interest in the development of supramolecular catalysts for various applications [12,13,14,15,16,17,18,19,20,21], including energy-related functions [22,23,24,25,26,27], we report here an efficient system for the photo-production of molecular hydrogen from water by associating TiO_2_ with carbon nanotubes and gold nanoparticles, to bring together the “best of three worlds”. Furthermore, due to the entangled nature of the carbon nanotube network, the CNT-based catalytic system can be integrated into a packed-bed microfluidic photoreactor [28] for the continuous flow production of H_2_. The latter approach combines the advantages of a nanostructured photocatalyst with the high surface-to-volume ratio of the microfluidic device, which will allow light to penetrate deep into the reaction medium for improved efficacy.

## 2. Materials and Methods

### 2.1. General

Unless otherwise specified, all chemicals were purchased from Sigma-Aldrich. Multi-walled carbon nanotubes (ref. NC700) were donated by Nanocyl (Sambreville, Belgium). Degussa P25 titanium dioxide nanopowder (21 nm) consists of an 80:20 mixture of rutile/anatase phases. DANTA was synthesized according to our previously reported procedure [29]. The synthesis of gold nanoparticles was adapted from methods detailed in previous literature [30]. The upper part of the batch photoreactor is made of a Pyrex glass tube, and the bottom part consists of a standard quartz cuvette with a 1 cm path length (ref. 221-10-40, Hellma—Paris, France). The valve of the batch reactor (ref. 90-04D) was purchased from CTVM (Collégien, France). The lamp used for photocatalysis in batch is a Xenon arc lamp (Oriel—500 W power, polychromatic light 200‒800 nm) and the lamp for continuous flow photocatalysis a Mercury-Xenon arc lamp (Hamamatsu LC8 01A—250 W, polychromatic light 200–600 nm) guided by an optical fiber.

### 2.2. Assembly of Gold Nanoparticles on Carbon Nanotubes (AuCNT)

The preparation of the AuCNT hybrid was carried out according to our previously reported procedure [31]. Briefly, we used a layer-by-layer approach to decorate carbon nanotubes, starting with the deposition of an anionic diacetylene nitrilotriacetic amphiphile (DANTA). Upon sonication with carbon nanotubes in water, DANTA molecules self-organize into nanoring-like structures by adsorption of hydrophobic chains on the CNT surface, with hydrophilic anionic heads oriented towards the aqueous medium. The nanorings are then stabilized by photopolymerization at 254 nm of the diacetylene units. A second layer is thereafter deposited by the addition of a cationic polymer (PDADMAC), which is immobilized by electrostatic interactions with the anionic head groups of DANTA. The cationic polymer serves as an interface between the nanotubes and the metal nanoparticles that will be embedded in the tri-dimensional polyammonium network. Freshly prepared gold nanoparticles (AuNP) were finally added to the multilayer assembly to provide access to AuCNT.

### 2.3. Characterization of the Hybrids

Inductively coupled plasma mass spectrometry (ICP-MS): AuCNT samples were mineralized at room temperature for 16 h using *aqua regia*. The samples were diluted 1000 times in ultrapure water, then 100 times in 2% HCl and injected at a flow rate of 100 µL min^−1^. Nebulization of the samples was performed by means of a microconcentric nebulizer. A 7700 × ICP-MS (Agilent, Santa Clara, CA, USA) was used as the elemental detector. The quantification of gold was performed at *m*/*z* = 197 using external standards ([Au] = 6 mM).

Transmission Electron Microscopy (TEM): grids were observed on a Philips CM12 microscope operated at 80 kV.

### 2.4. Microfluidic Device Fabrication

The fabrication of the microfluidic photoreactor was based on a standard PDMS soft-lithography replica molding. First, a Si/SU8 master mold was prepared by a two-layer photolithography, as the channels had different heights. SU8 photoresist (Chimie Tech, Antony, France) was spin coated onto a 4-inch Si wafer (Sil’Tronix Silicon Technologies, Archamps, France) using a spin coater (SPIN150, SPS-France, Vaulx-Milieu, France) as follows: SU8-2005 at 2500 rpm for the first layer and SU8-2100 at 1500 rpm for the second layer. To create the microstructures, Si/SU8 and the photomask were exposed to UV light (UV-KUB, KLOE, Saint-Mathieu-de-Tréviers, France). The photomask was created beforehand by designing the photoreactor with a CAD program (Klayout) and printing it on a polyester film (JD Photo Data, Hitchin, UK). The next step consisted of replicating the mold using polydimethylsiloxane (PDMS). Sylgard 184 prepolymer base was mixed with a curing agent (Neyco, Vanves, France) in a 10:1 ratio, poured onto the mold, and heated at 60 °C for 2 h. Once reticulated, the PDMS, which contained the microstructures, was peeled off the mold. Next, the inlet and outlet of the channels were pierced with a biopsy puncher (1.5 mm, VWR), and the chip was bonded/sealed on another flat piece of PDMS by oxygen plasma treatment (1 min, CUTE, Femto Science Inc., Hwaseong, Republic of Korea). The photoreactor was heated at 60 °C overnight for hydrophobic recovery.

### 2.5. Photocatalytic Reactions in Batch

With TiO_2_: A stock suspension of P25 titanium dioxide in water (10 mg mL^−1^) was sonicated in an ultrasonic bath for 1 h. Part of this suspension (200 µL) was transferred to the photoreactor containing an 80:20 water/MeOH mixture (final volume = 2 mL; [TiO_2_] = 1 mg/mL). The photocatalytic mixture was degassed for 30 min using argon bubbling, followed by a vacuum/backfill with argon cycles. This operation was repeated five times to remove the oxygen from the reactor. The photoreactor was placed 5 cm from the lamp and exposed to light for 45 min, with stirring. Following illumination, the reactor was connected to a gas chromatography instrument (Agilent μGC-R3000, SRA Instruments, Marcy l’Etoile, France) to measure the amount of hydrogen gas produced.

With CNT/TiO_2_: A stock suspension of CNT (10 mg mL^−1^) in water/methanol 80:20 was sonicated (bath) for 1 h. CNT suspension (200 µL) was added to the photoreactor containing TiO_2_/H_2_O/MeOH, previously exposed to a first cycle of irradiation for 45 min. The photocatalytic mixture was processed as above before the photoreactor was positioned 5 cm from the lamp and exposed to light for 45 min, with stirring. Hydrogen gas was measured by gas chromatography.

With AuCNT/TiO_2_: The suspension of AuCNT in water was freeze-dried. The dried nanohybrid was used to prepare a stock solution (10 mg mL^−1^) in water/methanol (80:20). AuCNT suspension (200 µL) was added to the photoreactor containing TiO_2_/H_2_O/MeOH, previously exposed to a first cycle of irradiation for 45 min. The photocatalytic mixture was processed as above before the photoreactor was positioned 5 cm from the lamp and exposed to light for 45 min, with stirring. Hydrogen gas was measured by gas chromatography.

Aggregates of P25-CNT and P25-AuCNT, obtained after the irradiation cycle, were used as photocatalysts for incorporation into the microfluidic device.

### 2.6. Photocatalytic Reactions under Microfluidic Conditions

#### 2.6.1. Loading of the TiO_2_/CNT or TiO_2_/AuCNT Catalyst

The above suspensions containing TiO_2_/CNT or TiO_2_/AuCNT (recovered after the batch reaction) were freeze-dried. The freeze-dried powder (200 µg) was suspended in water/methanol 80:20 (200 µL) and introduced in the microfluidic reservoir connected to the microreactor through PTFE tubing (1.5 mm diameter). The flow was established with a flow control system (FLOW EZ from Fluigent, Le Kremlin-Bicêtre, France) using ultra-pure argon. By applying pressure inside the reservoir (200 mbar), the nanohybrids were passed through the entry tube and trapped inside the microreactor, forming the catalytic packed-bed.

#### 2.6.2. Typical Experimental Procedure for the Hydrogen Production Reaction

An 80:20 water/methanol mixture (1 mL) was degassed beforehand by bubbling argon in the microfluidic reservoir for 30 min. This mixture was then injected at a flow rate of 8 μL min^−1^ into the device. The optical fiber connected to the lamp was positioned 3 cm from the microreactor. The effluent mixture was recovered at the outlet of the microreactor and collected in a sealed (rubber septum) 5 mL flask. The gas atmosphere in the flask was analyzed by gas chromatography (Shimadzu GC-2010 Plus, Kyoto, Japan). The gas phase was sampled through the septum using a Hamilton^®^ SampleLock syringe and injected immediately into the GC apparatus (Column: Carboxen 1010 Plot fused silica capillary column (30 m × 0.53 mm × 30 μm); injection temperature: 230 °C; column temperature: 150 °C; flow: 5 mL min^−1^; purge: 2 mL min^−1^; split ratio: 5.0; carrier gas: argon; detector: TCD 230 °C, 30 mA).

## 3. Results

### 3.1. Photocatalytic Hydrogen Production in Batch with TiO_2_ and TiO_2_/CNT

Commercially available Degussa P25 titanium dioxide was selected as the photo-catalyst to be used as a reference semi-conductor material to serve as the benchmark for the performance of our multicomponent catalytic system. TiO_2_ was used, either alone, or in combination with carbon nanotubes and gold. In the first set of experiments, TiO_2_ powder was introduced in the batch photo-reactor containing an 80:20 water/methanol mixture. Methanol was used here as a sacrificial reagent that is consumed by two distinct processes: (i) alcohol reformation, in which the photogenerated holes combine with the alcohol, affording H_2_ and CO_2_ [CH_3_OH → HCOH (+ H_2_) + H_2_O → HCO_2_H (+ H_2_) → CO_2_ (+ H_2_)], and (ii) O_2_ scavenging, affording H_2_O and CO_2_ [32,33]. The mixture was exposed to the xenon lamp for 45 min, with vigorous stirring. The photoreactor was then connected to a gas chromatograph and the amount of hydrogen produced was measured. Under these reaction conditions, 1497 µmol of H_2_ per gram of TiO_2_ per hour (µmol g^−1^ h^−1^) was generated upon photo-activation of TiO_2_, along with some carbon dioxide (ca. 9 µmol g^−1^ h^−1^), originating from the oxidation of methanol. Of note, the same batch of TiO_2_ catalyst could be reused for three additional photocatalytic runs of 45 min, affording a steady hydrogen gas production with a standard deviation of ± 133 µmol g^−1^ h^−1^.

**Figure 1:**
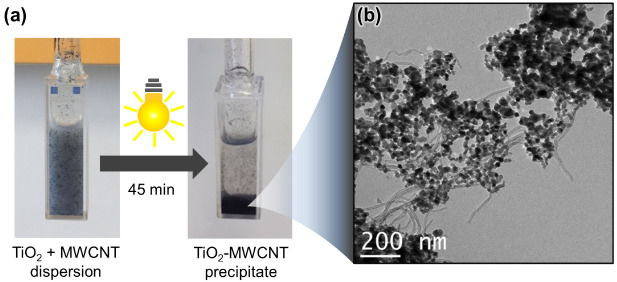
(**a**) Photoreactor before (left) and after (right) exposure to light; (**b**) TEM picture of TiO_2_/CNT aggregates.

After the first run, carbon nanotubes were introduced in the reactor containing TiO_2_, and the photocatalytic experiment was repeated. Thus, multi-walled CNT were added to the above suspension of TiO_2_ in an 80:20 H_2_O/MeOH mixture. The mixture was vigorously stirred under xenon lamp irradiation for 45 min, and the hydrogen production was assessed. In the presence of carbon nanotubes and TiO_2_, an increased hydrogen production was recorded at 12,273 µmol g^−1^ h^−1^, along with 100 µmol g^−1^ h^−1^ of CO_2_. These values show an 8-fold increase in hydrogen production, compared to experiments conducted with TiO_2_ under the same reaction conditions, but without CNT (1497 vs. 12,273 µmol g^−1^ h^−1^). A control experiment, conducted with CNT in the absence of TiO_2_, led to no hydrogen evolution, suggesting that the carbonaceous platform has an activating effect on TiO_2_ [34]. In addition, a macroscopic change in the appearance of the CNT/TiO_2_ mixture was observed after irradiation. In fact, the mixture was originally bluish in color, with some TiO_2_ suspended in the medium, but the cloudy suspension became colorless and a black solid precipitated rapidly after the first round of irradiation (Figure 1a). This change in the aspect of the CNT/TiO_2_ mixture was attributed to an aggregation phenomenon of TiO_2_ with carbon nanotubes. The formation of aggregates was further evidenced by transmission electron microscopy (TEM, Figure 1b) of the irradiated sample, which showed carbon nanotubes in intimate contact with TiO_2_. Although the exact nature of the interaction has not been elucidated, one can speculate that heterojunctions could be created at the interface of the semi-conductor nanoparticles and carbon nanotubes, resulting in a nanohybrid material with potential charge-dissociation properties. TEM pictures show that nanotubes form an entangled network supporting TiO_2_. This peculiar structuration of the nanohybrid photocatalyst makes it suitable for integration, as a packed-bed, in the restriction zone of a microfluidic device that would enable the continuous flow photoproduction of hydrogen. The TiO_2_/CNT nanohybrid was thus further investigated under microfluidic conditions. 

### 3.2. Microfluidic Reactions with TiO_2_/Carbon Nanotubes

#### 3.2.1. Design of the Microfluidic Photoreactor

From an engineering perspective, packed-bed microfluidic systems have restrictions that trap the catalyst and create the porous phase in which the transformation is taking place. The restriction zone will force the nanotube-based hybrids to aggregate and form an entangled porous material, while allowing the water/methanol solution to flow through, under irradiation [35]. This clustering effect of the CNT will be used to hold the photocatalytic nanohybrid in the device.

**Figure 2:**
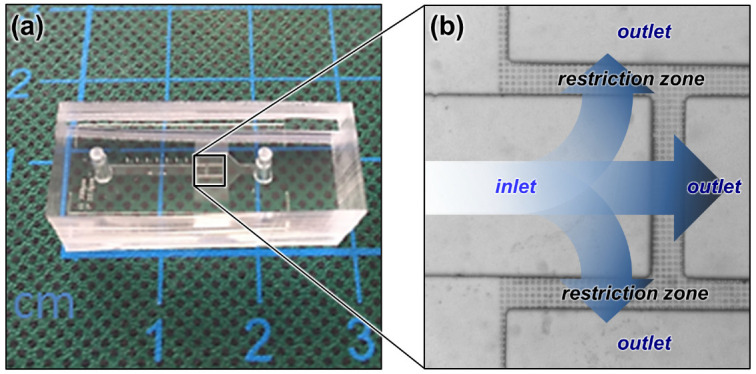
(**a**) Microfluidic photoreactor photograph; (**b**) optical microscopy image of the restriction zone.

The microfluidic device was fabricated with a restriction zone made of regularly spaced (15 µm) pillars (20 µm diameter × 6 μm height) intercalated in between the inlet and outlet channels (1000 µm width × 200 µm height). A standard polydimethylsiloxane (PDMS) soft-lithography replica molding technique was used for creating the microstructures and the channels (Figure 2a). PDMS was selected here as the polymer material, as it is transparent above ca. 300 nm, thus allowing activation of TiO_2_, whose absorbance extends up to ca. 380 nm. In order to increase the flow rate in the chip, the restriction zone was connected to three channels merging into a single outlet channel (Figure 2b).

#### 3.2.2. Loading of the Microreactor with the TiO_2_/CNT Nanohybrid

A catalytic packed-bed was created at the restriction zone by passing through 200 µg of the suspension of TiO_2_/CNT nanohybrid catalyst (the one obtained after a first round of irradiation in the batch reactor) in water/methanol. The final volume of the packed-bed was 0.8 µL. The stability of the nanohybrid was evaluated by washing the packed-bed with a mixture of methanol/water. ICP-MS analysis of the collected solvent phase showed no leaching of TiO_2_ from the device, confirming the robustness of the assembly and its resistance to the flow of solvents.

#### 3.2.3. TiO_2_/CNT Nanohybrid-Photocatalyzed Continuous Flow Production of Hydrogen

**Figure 3:**
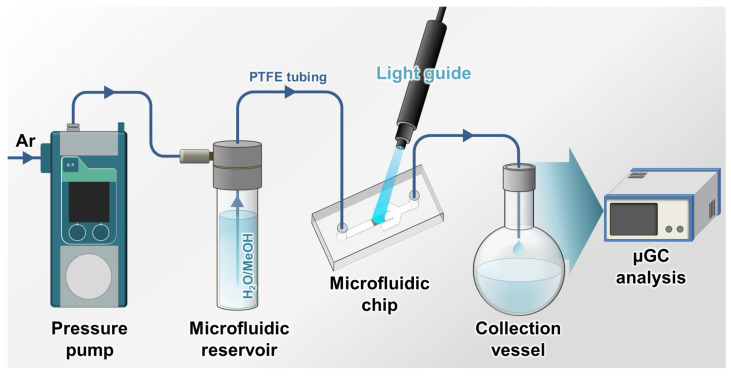
Setup used for the investigation of H_2_ production under microfluidic conditions.

As the dimensions of the xenon lamp that was used for the initial batch reactions were not compatible with the microreactor setup, another light source had to be used. We thus selected a mercury-xenon lamp coupled to an optical fiber to guide the light beam towards the chip. The TiO_2_/CNT catalytic packed-bed was then exposed to light, and a degassed 80:20 water/methanol solution was injected at a flow rate of 8 µL min^−1^. The setup was pressurized with 200 mbar of argon applied to the inlet, allowing the solution to pass through the PTFE tubing and the chip (Figure 3). The irradiated solution was collected in a sealed flask and hydrogen gas production was measured at the end of the experiment by gas chromatography.

A hydrogen production of 2192 µmol g^−1^ h^−1^ was observed under these reaction conditions, with a contact time between the solution and the catalyst of 6 seconds. However, direct comparison with the batch reaction is not straightforward, as the setup is different. Nevertheless, the photo-activation of TiO_2_/CNT in the microfluidic device allowed hydrogen to be continuously generated and gradually evacuated with the liquid flow. The chip was used over three runs, with no alteration of its performances, affording consistent hydrogen production with a standard deviation of ± 223 µmol g^−1^ h^−1^ (2551 µmol g^−1^ h^−1^ for the 2nd run, 2144 µmol g^−1^ h^−1^ for the 3rd run).

### 3.3. Photocatalyzed Hydrogen Production with TiO_2_/Gold/Carbon Nanotubes

In order to achieve more efficient photocatalysis, we next proceeded to combine TiO_2_/CNT with gold nanoparticles. As previously mentioned in the introductory section, the association with metallic nanoparticles could favor charge separation. In addition, light activation of the plasmonic resonance of gold could also be beneficial to the photocatalytic process.

#### 3.3.1. Assembly of Gold Nanoparticles on Carbon Nanotubes

**Figure 4:**
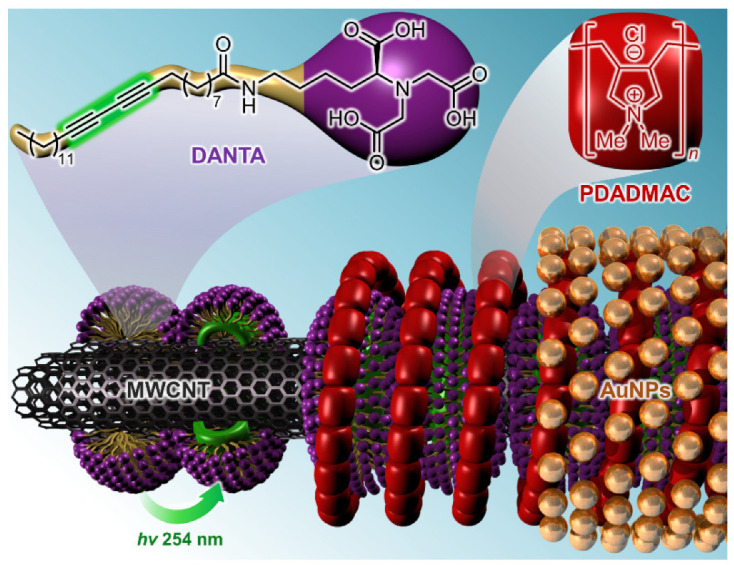
Illustration of the assembly of gold nanoparticles on a carbon nanotube using a layer-by-layer strategy.

The assembly of gold nanoparticles (AuNP) on the CNT surface was achieved by a process previously developed in our group (Figure 4) [31] using a layer-by-layer approach consisting of: (i) the addition of a polymerizable amphiphile (DANTA) to the nanotube, (ii) photopolymerization, (iii) the addition of a cationic polymer (PDADMAC), and (iv) the addition of AuNP to the multilayer assembly. The AuCNT hybrid was obtained as an aqueous dispersion, and the gold concentration was measured by ICP-MS ([Au] = 6 mM).

#### 3.3.2. Photocatalyzed Hydrogen Production in a Batch Reactor with TiO_2_/Gold/Carbon Nanotubes

**Figure 5:**
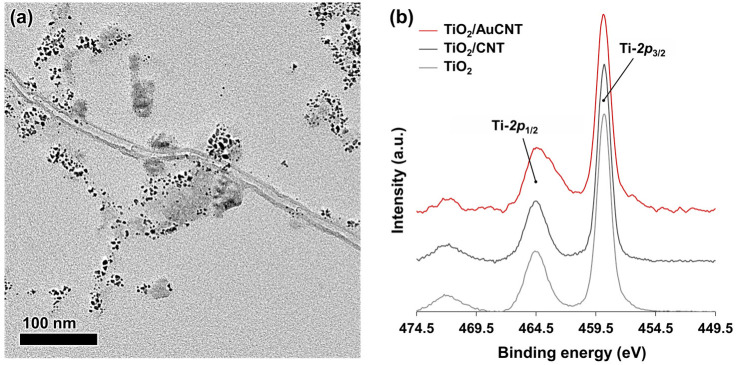
(**a**) TEM picture of TiO_2_/AuCNT; (**b**) XPS analyses of TiO_2_, TiO_2_/CNT, and TiO_2_/AuCNT.

The AuCNT aqueous suspension was lyophilized, and the catalyst powder was introduced in the batch photo-reactor containing an 80:20 water/methanol mixture and TiO_2_. The mixture was exposed to the xenon lamp for 45 min, with continuous stirring leading to a TiO_2_/AuCNT hybrid (Figure 5a). The photo-reactor was then connected to GC to quantify the produced hydrogen. Under these reaction conditions, a hydrogen production of 30,430 µmol g^−1^ h^−1^ was detected upon photo-activation of TiO_2_/AuCNT, along with 248 µmol g^−1^ h^−1^ of carbon dioxide. This hydrogen production value must be compared to those of the experiments previously conducted with TiO_2_ (1497 µmol g^−1^ h^−1^) and TiO_2_/CNT (12,273 µmol g^−1^ h^−1^). The AuCNT-containing system allowed a 2.5-fold increase in hydrogen production, compared to that of TiO_2_/CNT, and a spectacular 20-fold increase, compared to that of TiO_2_ only. A control experiment, conducted with AuCNT in the absence of TiO_2_, led to a limited hydrogen production of 540 µmol g^−1^ h^−1^. The latter experiment indicates that the contribution of AuCNT goes beyond a cumulative effect, as the hydrogen production of TiO_2_/AuCNT (i.e., 30,430 µmol g^−1^ h^−1^) is greater than the sum of the hydrogen production of TiO_2_/CNT and AuCNT (i.e., 12,273 µmol g^−1^ h^−1^ and 540 µmol g^−1^ h^−1^, respectively) when studied separately. There is thus a synergistic effect of the nanohybrid on the hydrogen production yield. An additional control experiment was carried out by simply adding gold nanoparticles to the TiO_2_/CNT system. In that case, only a minor increase in the H_2_ production was observed (from 12,273 µmol g^−1^ h^−1^ for TiO_2_/CNT to 13,851 µmol g^−1^ h^−1^ after addition of AuNP), indicating that structuration of gold near the surface of carbon nanotubes plays an important role in the process.

#### 3.3.3. Flow Reaction with TiO_2_/AuCNT

Since the TiO_2_/AuCNT combination showed improved activity in the photo-catalyzed hydrogen production, we next proceeded with the integration of the new catalytic system in the microfluidic device.

As developed above for TiO_2_/CNT, a photocatalytic packed-bed was created at the restriction zone of the microfluidic device by introducing 200 µg of TiO_2_/AuCNT (as a suspension in H_2_O/MeOH) obtained after the first round of irradiation in a batch reactor. The final volume of the nanohybrid packed-bed was 0.8 µL. The stability of the catalytic packed-bed was evaluated by washing with a methanol/water mixture. ICP-MS analysis of the collected solvent phase showed neither leaching of TiO_2_ nor gold (<1 ppb) from the device, confirming the robustness of the assembly. The TiO_2_/AuCNT catalytic packed-bed was thereafter exposed to light before an 80:20 water/methanol solution was injected at a flow rate of 8 µL min^−1^. A hydrogen production of 5462 µmol g^−1^ h^−1^ was observed under these reaction conditions, with a contact time of 6 seconds. Under flow reaction conditions, TiO_2_/AuCNT produces 2.5 times more hydrogen, compared to TiO_2_/CNT. This enhancement in hydrogen production under flow conditions is in the same range as the enhancement observed under batch conditions upon the addition of AuCNT.

## 4. Discussion

The addition of CNT or AuCNT to TiO_2_ was shown to be beneficial to the photocatalyzed hydrogen production reaction. Under batch conditions, compared to TiO_2_ alone, an amplification of H_2_ production by a factor 8 and 20 was observed upon the addition of CNT or AuCNT, respectively (Table 1). The same trend was observed under flow reaction conditions (TiO_2_/CNT vs. TiO_2_/AuCNT). However, TiO_2_ could not be tested alone as a reference material in flow, since without the CNT support, its incorporation/retention in the device could not be achieved.

We hypothesize that, upon photo-activation, carbon nanotubes contribute to the dissociation of electron–hole pairs in TiO_2_. This dissociation minimizes the non-productive recombination of charges. Although it is commonly accepted that multiwalled nanotubes can act as electron acceptors when combined with photoactive compounds [36], a recent publication reports that ultrafast (<1 ps) hole transfer can also occur at the interfaces between CNT and TiO_2_ [37]. This transfer leads to an accumulation of electrons in the conduction band of TiO_2_, providing a high density of active sites for the photo-dissociation of the water/methanol mixture and hydrogen production. Due to charge separation between the nanotube and TiO_2_, the kinetics of the electron–hole recombination is minimized. 

The increased performance of TiO_2_/AuCNT, compared to TiO_2_/CNT, can be rationalized by some additional effects of gold that are mediated by gold–titanium dioxide interactions. XPS analysis of TiO_2_ displayed two characteristic peaks at binding energies of 464.4 eV for Ti-*2p*_1/2_, and 458.8 eV for Ti-*2p*_3/2_ (Figure 5b). Although no drastic shifts were detected in the XPS survey of the nanohybrids (TiO_2_/CNT and TiO_2_/AuCNT), the peaks are broader, suggesting partial charge transfer (see also Figure S1, in the Supplementary Material, for a comparison of UV-vis diffuse reflectance spectroscopy profiles of the different catalytic materials). Another likely effect of the CNT is its role as a dispersing agent, which prevents bonded TiO_2_ from agglomerating, thus providing a higher active surface area. The Xe lamp emits broad-spectrum wavelengths covering UV and visible regions. In the UV region, the electrons produced by the photo-excitation of TiO_2_ can migrate and accumulate at the surface of gold nanoparticles. This accumulation would be concomitant with the migration of holes towards the nanotubes, as mentioned above. In the visible region, plasmonic gold is able to sensitize TiO_2_ and inject additional “hot” electrons into the conduction band of the semiconductor material (i.e., TiO_2_). This process leaves holes in the noble metal nanoparticles that could also migrate to the CNT, for improved charge separation. Alternatively, the charge transfer process can be reverted, so that carbon nanotubes act as electron acceptors, as also reported in the literature [38,39,40].

The association of TiO_2_ with gold and carbon nanotubes [41,42] for photocatalytic applications has previously been investigated by others, not for H_2_ production, but rather for the degradation of pollutants. On the other hand, the combination of TiO_2_ with carbon nanotubes [43] or gold [44] has been studied for the photocatalyzed production of hydrogen. Yet, no integration of TiO_2_/CNT in microfluidic devices has been reported so far. TiO_2_-based microreactors for photocatalytic water splitting rely, for example, on planar microreactors incorporating Pt/TiO_2_ photocatalyst thin films [45,46] that lead to a hydrogen production rate of 766 µmol g^−1^ h^−1^ under a simulated solar light irradiation. In other examples, the loading of Pt/TiO_2_ photocatalyst was achieved by a casting-transfer method on the PDMS matrix constituting the walls of the microreactor, or by the coating of the micro-pillars [47] in the reaction micro-chamber. Compared to the abovementioned systems, our packed-bed assembly is more straightforward, as it requires neither specific treatment of the channels, nor in situ deposition of the photocatalyst. This work represents the first example of successful packed-bed integration of TiO_2_/CNT and TiO_2_/Au/CNT in a microfluidic chip whose design was optimized to ensure an optimal flow of water/methanol and pressure distribution. The chip offers some advantages in terms of implementation (on-demand production of H_2_), practicability (no final catalyst removal from the reaction mixture), safety (no hydrogen gas build-up), and recyclability (reuse of the chip). In addition, the setup also provides a suitable answer to the main challenges faced by conventional batch TiO_2_ reactions, i.e., optimal light distribution, the recombination of photo-generated electron–hole pairs, and mass transfer.

## 5. Conclusions

In this work, we achieved the first integration of TiO_2_/CNT and TiO_2_/Au/CNT as photoactive catalytic packed-beds in microfluidic reactors. The produced chips were investigated in the production of hydrogen from water/methanol. The peculiar structuration of the entangled CNT network permitted efficient retention of the photocatalysts in the microfluidic device. The association of TiO_2_ with CNT and gold permitted the significant improvement of hydrogen production thanks to: (i) the stabilization of the photo-generated electron–hole pairs from TiO_2_ and charge delocalization towards the carbon nanotubes, and (ii) the activation of gold in the visible region and the creation of additional “hot” electrons that can be transferred to TiO_2_. TiO_2_, gold, and carbon nanotubes work in a synergistic fashion, leading to improved performances. It is anticipated that the same strategy could be applied to other metals/semi-conductor materials for application in related fields such as the photocatalytic degradation of pollutants. As such, CNT represent a highly versatile option towards nano-photocatalysis approaches under microfluidic conditions.

## Figures and Tables

**Table 1:** Photocatalyzed hydrogen production with different TiO_2_-based systems, in batch and in flow.

Entry	Catalyst	Batch ^1^		Flow ^2^	
		Time (min)	H_2_ Production (µmol g^−1^ h^−1^)	Contact Time (s)	H_2_ Production (µmol g^−1^ h^−1^)
1	TiO_2_	45	1497	N/A	N/A
2	TiO_2_/CNT	45	12,273	6	2192
3	TiO_2_/AuCNT	45	30,430	6	5462

^1^ Reaction conditions: TiO_2_ (2 mg) + CNT (2 mg) or AuCNT (2 mg), if applicable—2 mL H_2_O/CH_3_OH (80:20)—500 W Xe lamp; ^2^ Reaction conditions: 200 µg of TiO_2_/CNT or TiO_2_AuCNT—1 mL H_2_O/CH_3_OH (80:20)—250 W Hg-Xe lamp.

## Data Availability

The data presented in this study are available in article.

## Supplementary Materials

The following supporting information can be downloaded at: https://www.mdpi.com/article/10.3390/nano13071184/s1, Figure S1: Diffuse reflectance UV-visible spectra of TiO_2_, TiO_2_/CNT, and TiO_2_/AuCNT.
