# Supplementary material for: Continuous Flow Photocatalytic Hydrogen Production from Water Synergistically Activated by TiO2, Gold Nanoparticles, and Carbon Nanotubes
Source: Nanomaterials (Basel). 2023 Mar 27;13(7):1184. doi: 10.3390/nano13071184 (PMC10097087; doi:10.3390/nano13071184)
Supplement: Supplementary file 1 [file nanomaterials-13-01184-s001.zip › nanomaterials-2213128-supplementary.pdf]

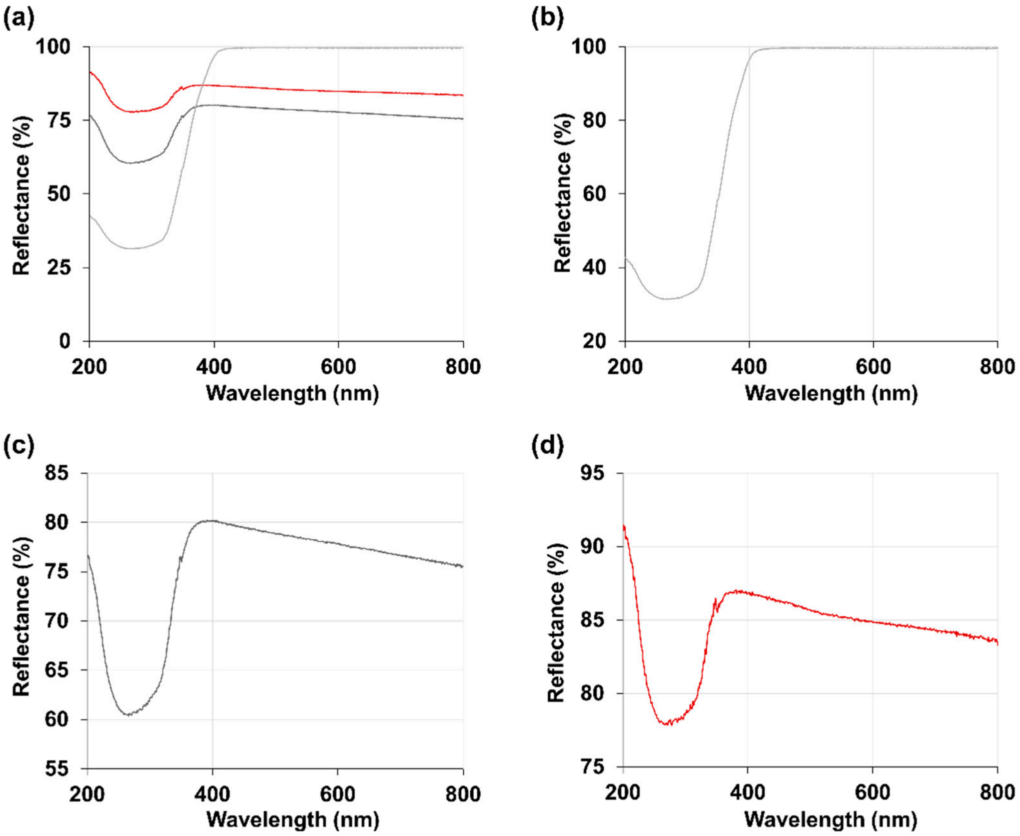

Figure S1. (**a**) Diffuse reflectance spectra of TiO_2 (grey curve), TiO_2/CNT (dark grey curve), TiO_2/AuCNT (red curve) and close-ups corresponding to (**b**) TiO_2, (**c**) TiO_2/CNT, and (**d**) TiO_2/AuCNT. All powders were prepared as 1 wt% dispersions in $BaSO_4$ and spectra were corrected using the diffuse reflectance of pure $BaSO_4$ as reference.
